# Hypertension and Risk of Cataract: A Meta-Analysis

**DOI:** 10.1371/journal.pone.0114012

**Published:** 2014-12-04

**Authors:** Xiaoning Yu, Danni Lyu, Xinran Dong, Jiliang He, Ke Yao

**Affiliations:** 1 Eye Center, Second Affiliated Hospital, School of Medicine, Zhejiang University, Hangzhou, China; 2 Institutes of Environmental Medicine, School of Medicine, Zhejiang University, Hangzhou, China; 3 Zhejiang Provincial Key Laboratory of Ophthalmology, Hangzhou, China; The Chinese University of Hong Kong, Hong Kong

## Abstract

**Background:**

Cataract is the major cause of blindness across the world. Many epidemiologic studies indicated that hypertension might play an important role in the development of cataract, while others not. We therefore conducted this meta-analysis to determine the relationship between risk of cataract and hypertension.

**Methods:**

Retrieved studies on the association of hypertension with cataract risk were collected from PubMed, Web of Science and the Cochrane Library during June 2014 and were included into the final analysis according to the definite inclusion criteria. Odds ratio (OR) or risk ratio (RR) were pooled with 95% confidence interval (CI) to evaluate the relationship between hypertension and cataract risk. Subgroup analyses were carried out on the basis of cataract type, race and whether studies were adjusted for main components of metabolic syndrome (MS).

**Results:**

The final meta-analysis included 25 studies (9 cohort, 5 case-control and 11 cross-sectional) from 23 articles. The pooled results showed that cataract risk in populations with hypertension significantly increased among cohort studies (RR 1.08; 95% CI: 1.05–1.12) and case-control or cross-sectional studies (OR 1.28; 95% CI: 1.12–1.45). This association was proved to be true among both Mongolians and Caucasians, and the significance was not altered by the adjustment of main components of MS. Subgroup analysis on cataract types indicated that an increased incidence of posterior subcapsular cataract (PSC) resulted among cohort studies (RR 1.22; 95% CI: 1.03–1.46) and cross-sectional/case-control studies (OR 1.23; 95% CI: 1.09–1.39). No association of hypertension with risk of nuclear cataract was found.

**Conclusions:**

The present meta-analysis suggests that hypertension increases the risk of cataract, especially PSC. Further efforts should be made to explore the potential biological mechanisms.

## Introduction

Cataract has been a major cause of visual impairment among senior citizens worldwide [1]. According to data provided by the World Health Organization (WHO), cataract is responsible for nearly 50% of blindness across the world [2]. With the coming of aging society, the prevalence of cataract increases rapidly. The importance of risk factors identification for cataract is therefore evident. In the past decades, researchers have performed numerous in-depth epidemiologic studies to understand the pathogenesis of cataract [3]–[11], many of which indicated that hypertension plays an important role in the development of cataract [5], [8]–[9], [11].

Hypertension is considered to cause elevation of inflammatory cytokines such as tumor necrosis factor-alpha (TNF-α), interleukin-6(IL-6) [12]. Besides, an elevation of C-reactive protein (CRP) level has been detected when individual blood pressure raises [12]–[13]. Considering that cataract is closely related to intense systemic inflammation [14]–[15], hypertension is therefore involved in the pathological pathway of cataract development through an inflammatory mechanism. Beyond that, Lee et al. [16] reported that hypertension could induce conformation structure alteration of proteins in lens capsules, thereby exacerbating the cataract formation. Although several plausible mechanisms have been proposed based on laboratory results, the conclusions from epidemiologic studies remain inconsistent. It is important to determine the effects of hypertension on cataract risk, due to increasing hypertension morbidity. Given the fact that individual studies may be limited because of sample size, therefore, a meta-analysis was conducted to quantitatively confirm the relationship between hypertension and cataract risk. What’s more, many scholars hold the opinion that hypertension might be linked to cataract by other main components of MS (pathoglycemia, obesity and dyslipidemia) [17]–[18], a subgroup analysis containing studies adjusted for these confounders will be helpful to figure out the truth.

## Materials and Methods

### Search Strategy and Study Selection

PubMed, Web of Science, and the Cochrane Library databases were searched for original articles, published between January 1990 and May 2014, on the relationship between hypertension and cataract risk. The search strategy included cataract (“cataract”, “lens opacities”, “crystalline opacities”), hypertension (“hypertension”, “high blood pressure”), and human studies. The references of selected papers were manually searched for potentially relevant new papers. The initial selection of studies was performed on the basis of titles and abstracts. Next, two investigators (XN Yu and DN Lyu) independently screened the full text of each selected study using the inclusion criteria. In the present meta-analysis, the included studies met the following inclusion criteria: (1) original research papers reporting independent data; (2) case-control, cross-sectional, or cohort studies estimating the influence of hypertension on cataract risk with odds ratio (OR) or relative ratio (RR) and its 95% confidence interval (CI); and (3) English articles published from January 1990 to May 2014. We excluded papers that were not age-adjusted, as age is considered the most reliable independent risk factor for cataract. To avoid double publication, only the most recent or most informative studies were included. This meta-analysis was performed in accordance with the Preferred Reporting Items for Systematic Reviews and Meta-Analyses (PRISMA) statement checklist [19].

### Data Extraction and Study Quality Assessment

The following data were extracted from the included studies: first author’s name, year of publication, country, study design, sample size, cataract definition, hypertension status, cataract subtypes (nuclear, cortical, and PSC), adjusted variables and OR/RR values with corresponding 95% CI. Only one model could be selected from studies with more than one adjustment model. We selected the model in which the OR/RR values were adjusted to the maximum extent for potentially confounding variables. Hypertension was diagnosed by study researchers or physicians. Standard diagnostic criteria for cataract included the Lens Opacities Classification System (LOCS)I–III, the Age-Related Eye Disease Study (AREDS), the Wisconsin Cataract Grading System and the Wilmer cataract grading system. The Newcastle-Ottawa scale (NOS) was used to evaluate the study quality [20]. The studies that met at least five NOS criteria were considered to be high quality studies.

Two authors (XN Yu and DN Lyu) independently determined whether each study met the aforementioned inclusion criteria; they then extracted the data and evaluated the study quality based on NOS criteria. Conflicting results were discussed among the study group and solved through consultation.

### Data Synthesis and Statistical Analysis

Stata version 12.0 software (Stata Corporation, College Station, TX) was used for statistical analysis in this meta-analysis. The significance levels were set to *P*<0.05 or *P*<0.01, except for heterogeneity. The OR/RR values with corresponding 95% CI served as the valid estimates for all qualified studies to obtain a pooled OR/RR. Two independent meta-analyses were performed according to protocol design. The subgroup analyses were based on cataract subtypes, race and confounders (main components of MS except hypertension: pathoglycemia, obesity and dyslipidemia). In the studies affording data only on single cataract subtype, the subtype-specific OR/RR value was treated as an estimate for any type of cataract. In studies reporting OR/RR of at least two subtypes, the method of Jan Hamling [21] was used for estimating the adjusted overall OR/RR on the basis of Greenland and Longnecker’s effective numbers approach. The method described by Zhang [22] was utilized for approximating an adjusted RR from the adjusted OR.

Potential heterogeneity among the individual studies was evaluated by means of Cochran’s Q statistic and I^2^ index score, with a significance set at the *P*-value <0.10 or I^2^ score >50% [23]. When no heterogeneity was detected among the included studies, the fixed-effects model (inverse variance method) was used; otherwise, the random-effects model (DerSimonian and Laird method) was used for reducing the errors [24]. A sensitivity analysis was performed to evaluate the robustness of the main meta-analysis results; and meta-regression analyses were conducted to explore the potential resources of heterogeneity. Potential publication bias was evaluated by Egger’s linear regression [25] and Begg’s rank correlation tests [26].

## Results

### Characteristics of Included Studies

**Figure 1:**
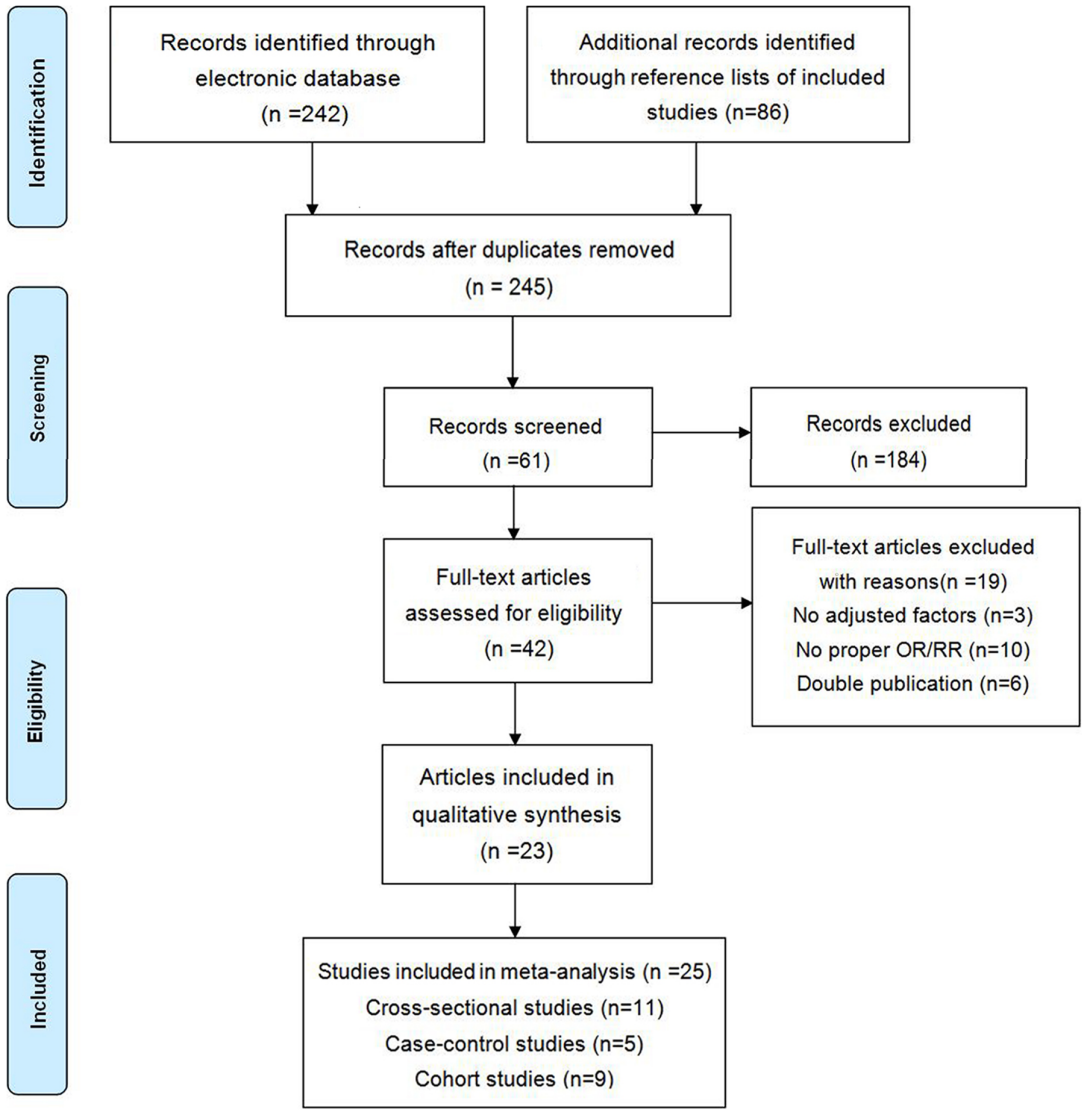
Flow diagram of study selection process.

The search strategy identified 245 unique articles, from which 42 full-text articles were retrieved for final review after screening titles and abstracts (Figure 1). Of these, 25 studies from 23 articles that met all predefined inclusion criteria were include in our meta-analysis [3]–[11], [17]–[18], [27]–[38], including 9 cohort studies, 5 case-control studies and 11 cross-sectional studies. The remaining 19 articles were excluded for the following reasons: 10 articles did not provide OR/RR or sufficient information to estimate OR/RR values [39]–[48], 3 articles provided crude OR instead of OR adjusted for age [49]–[51], and the data used in 6 articles had been used in other studies [52]–[57].

Characteristics of the included studies were summarized and shown in Table 1 and Table 2. One article including two different sets of data was extracted as two separate studies [27]. Another article including two separate sets of data according to gender was also considered as two studies [7]. Four studies that did not provide sufficient information to estimate an overall OR/RR for any type of cataract were used only in subgroup analysis [3], [5], [35], [38]. Some studies contained only females or males [17], [31], [33], while the others contained both genders. Data from the Visual Impairment Project, the French Age-Related Eye Diseases Prospective Study and the Blue Mountains Eye Study were used in both cohort and case-control/cross-sectional studies; and both study types were included in our analysis [3]–[4], [6], [11], [18], [32]. Because of the high incidence of cataract, OR is not equal to RR. Thus, OR was used in the analysis of cross-sectional and case-control studies, while RR was utilized only in the analysis of cohort studies. The criteria used to diagnose cataract were different from each other in some studies, and the cases in other studies were confirmed by medical record review. Based on the NOS system, the 23 articles were considered to be high-quality studies.

**Table 1:** Characteristics of 16 Case-Control/Cross-Sectional Studies Included into Present Meta-Analysis.

Source(Published Year, Country)	StudyDesign	Race	SampleSize	Age(year)	Cataracttypes	CataractDefinition	HTNDiagnosis	AdjustedVariables	NOS
**Wang** **(2009 China)**	Population-basedcross-sectional	Mongolians	3222	≥40	NC, CC, PSC	Standardcriteria	Confirmedby study researchers	Age, gender, region, glucose,income, BMI, education, et al.	8
**Delcourt** **(2000 France)**	Population-basedcross-sectional	Caucasians	2584	60–95	Any type	Standardcriteria	Confirmedby study researchers	Age, sex, diabetes,smoking	8
**Galeone** **(2010 Italy)**	Hospital-basedcase-control	Caucasians	2283	35–79	Any type	Medicalrecord review	Confirmedby study researchers	Age, central obesity, sex,diabetes, smoking, et al.	7
**Sabanayagam** **(2011 Singapore)**	Population-basedcross-sectional	Mongolians	2794	40–80	Any type, CC, NC, PSC	Standardcriteria	Confirmedby study researchers	Age, sex, BMI, diabetes,multivitamin use, smoking, et al.	8
**Leske** **(1999 Barbados)**	Population- basedcase-control	Melanoderm	4314	40–84	Any type, NC, CC, PSC	Standardcriteria	Confirmedby study researchers	Age, gender, waist/hipratio, diabetes	8
**Shah** **(2007 Pakistan)**	Population-basedcross-sectional	Mongolians	16402	≥30	Any type	Standardcriteria	Confirmedby study researchers	Age, sex	9
**Rim** **(2014 Korea)**	Population-basedcross-sectional	Mongolians	11591	≥40	Any type	Standardcriteria	Confirmedby study researchers	Age, sex, education,diabetes, sun exposure, et al.	8
**Nirmalan** **(2004 India)**	Population-basedcross-sectional	Mongolians	5140	≥40	Any type, NC, CC, PSC	Standardcriteria	Confirmedby study researchers	Age, diabetes, uric acid,waist-hip ratio, smoking, et al.	8
**Tavani** **(1995 Italy)**	Hospital-basedcase-control	Caucasians	1541	19–75	Any type	Medicalrecord review	None shown	Age, education, diabetes,BMI, clinical obesity, smoking, et al.	6
**Tsai** **(2003 China)**	Population-basedcross-sectional	Mongolians	1361	≥65	CC, NC, PSC	Standardcriteria	Confirmedby study researchers	Age, gender, diabetes,waist/hip ratio, smoking, et, al	8
**Ughade (1998 India)**	Hospital-basedcase- control	Mongolians	524	51–70	Any type	Lens opacityimpairing vision	Confirmedby study researchers	Age, diabetes, smoking,glaucoma, myopia, et al.	7
**McCarty** **(2000 Australia)**	Population-basedcase-control	Caucasians	4744	40–98	PSC	Standardcriteria	Confirmedby physicians	age, residence, refractivestatus	7
**Chen** **(2011 China)**	Population-basedcross-sectional	Mongolians	661	≥65	Any type	Medicalrecord review	Confirmedby physicians	age, diabetes, pulsepressure, homocysteine, folate, et al.	7
**Chen** **(2011 China)**	Population-basedcross-sectional	Mongolians	645	≥65	Any type	Medicalrecord review	Confirmedby physicians	age, diabetes, pulsepressure, homocysteine, folate, et al.	6
**Goodrich** **(1999 Australia)**	Population-basedcross-sectional	Caucasians	3654	49–97	CC, NC, PSC	Standardcriteria	Confirmedby study researchers	Age, sex, BMI, smoking,serum cholesterol, diabetes, et al.	7
**Machan** **(2012 Canada)**	Hospital-basedcross- sectional	Caucasians	6397	1–95	Any type, CC, NC, PSC	Standardcriteria	Confirmedby physicians	Age, sex, statin usesmoking, type 2 diabetes,	6

CC: cortical cataract; NC: nuclear cataract; HTN: hypertension; BMI: body mass index.

**Table 2:** Characteristics of 9 Cohort Studies Included into Present Meta-Analysis.

Source(Published Year, Country)	Studydesign	Race	SampleSize	Age(year)	Cataracttypes	CataractDefinition	HTNDiagnosis	Adjustedvariables	NOS
**Tan** **(2008 Australia)**	Population-based cohort	Caucasians	3654	≥49	Any type, NC,CC, PSC	Standardcriteria	Confirmed bystudy researchers	Age, diabetes, totalcholesterol, sex, et al.	9
**Schaumberg** **(2001 USA)**	Population-based cohort	Caucasians	17762	40–84	Any type, NC,CC, PSC	Medicalrecord review	Confirmed bystudy researchers	Age, diabetes, smoking,BMI, alcohol, et al.	6
**Lindblad** **(2008 Sweden)**	Population-based cohort	Caucasians	34595	49–83	Any type	Medicalrecord review	Confirmed bystudy researchers	Age, smoking,educational, diabetes, et al.	8
**Goldacre** **(2012 Britain)**	Hospital-based cohort	Caucasians	18646	0–80+	Any type	Medicalrecord review	Confirmed byphysicians	Sex, age, district ofresidence, et al.	6
**Goldacre** **(2012 Britain)**	Hospital-based cohort	Caucasians	208863	0–80+	Any type	Medicalrecord review	Confirmed byphysicians	Sex, age, region ofresidence, et al.	6
**Mukesh** **(2006 Australia)**	Population-based cohort	Caucasians	3721	43–84	NC, PSC	Standardcriteria	None shown	Age	8
**Klein** **(1998 USA)**	Population-based cohort	Caucasians	3683	≥40	NC, CC, PSC	Medicalrecord review	Confirmed bystudy researchers	Age, sex, smoking,heavy drinking	9
**Storey** **(2013 USA)**	Population-based cohort	Caucasians	2585	65–84	NC, CC	Standardcriteria	Confirmed byphysicians	Age, sex, smoking,alcohol status, et, al.	8
**Delcourt** **(France 2003)**	Population-based cohort	Caucasians	1947	≥60	Any type, NC,PSC, CC	Standardcriteria	Confirmed bystudy researchers	Age, smoking, education,BMI, diabetes, et al.	9

### Case-Control and Cross-Sectional Studies

Table 1 showed that 16 case-control and cross-sectional studies were included in the analysis, of which 14 studies were used in the analysis of relationship between hypertension and any type of cataract risk; 7 studies were used in the subgroup analysis based on cataract subtype; 5 studies were adjusted for pathoglycemia, obesity and dyslipidemia; 8 studies were conducted among Mongolians. 11 studies were population-based, and 5 were hospital-based.

**Figure 2:**
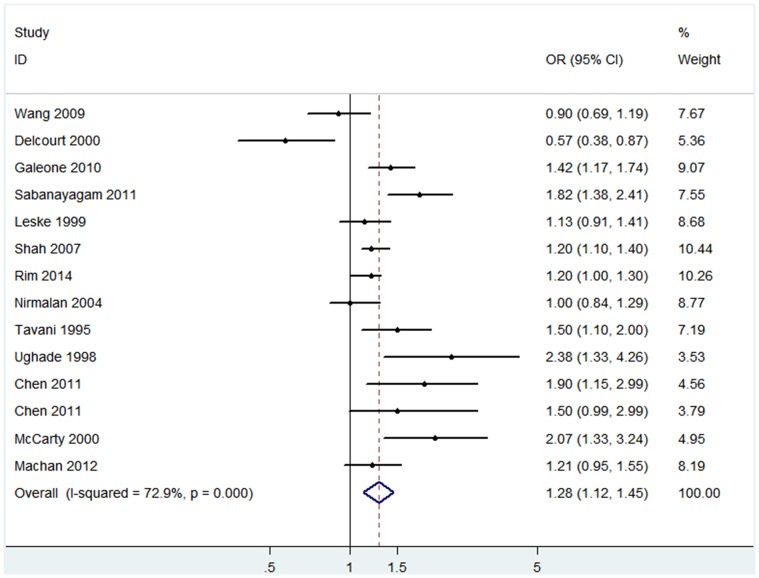
The association of hypertension with cataract risk in case-control/cross-sectional studies.

The pooled results of the 14 studies showed that risk of any type of cataract was significantly higher in patients with hypertension (OR 1.28, 95% CI: 1.12–1.45; I^2^ = 72.9%, *P_ heterogeneity_*<0.001) than in subjects with normal blood pressure (Figure 2). No significant publication bias was found among the included studies (Begg’s *P* = 0.23, Egger’s *P* = 0.34). Similar estimates were found among Mongolians (OR 1.30, 95% CI: 1.08–1.55; I^2^ = 77.7%, *P_ heterogeneity_*<0.001; Begg’s *P* = 0.54, Egger’s *P* = 0.26). Sensitivity analysis by sequentially omitting individual studies did not alter the significance of pooled OR estimates [3], [7]–[10], [28]–[32], [34], [36]–[37], which ranged from 1.24 (95% CI: 1.09–1.41) to 1.32 (95% CI: 1.17–1.48). We conducted meta-regression analyses to explore the influences of sample size, study design, study conducted race, publication year and cataract criteria on heterogeneity, but none of them was proved to be the main source of heterogeneity (*P*>0.05).

**Figure 3:**
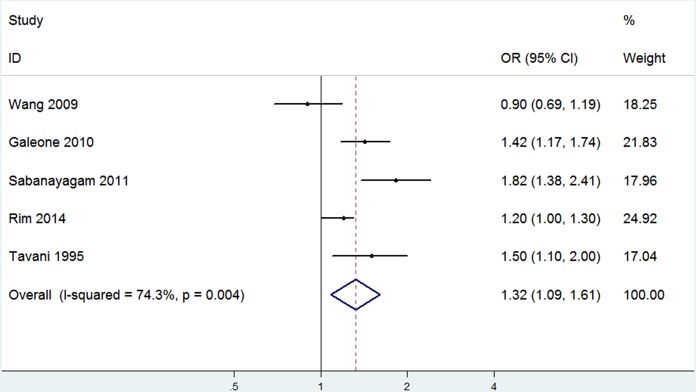
The association of hypertension with cataract risk after adjustment for MS components in case-control/cross-sectional studies.

Subgroup analysis of studies adjusted for pathoglycemia, obesity and dyslipidemia indicated that hypertension was independently related to an increased prevalence of cataract without the influence of its three complications (OR 1.32, 95% CI: 1.09–1.61; I^2^ = 74.3%, *P_ heterogeneity_* = 0.004; Begg’s *P* = 0.81, Egger’s *P* = 0.62) (Figure 3).

**Figure 4:**
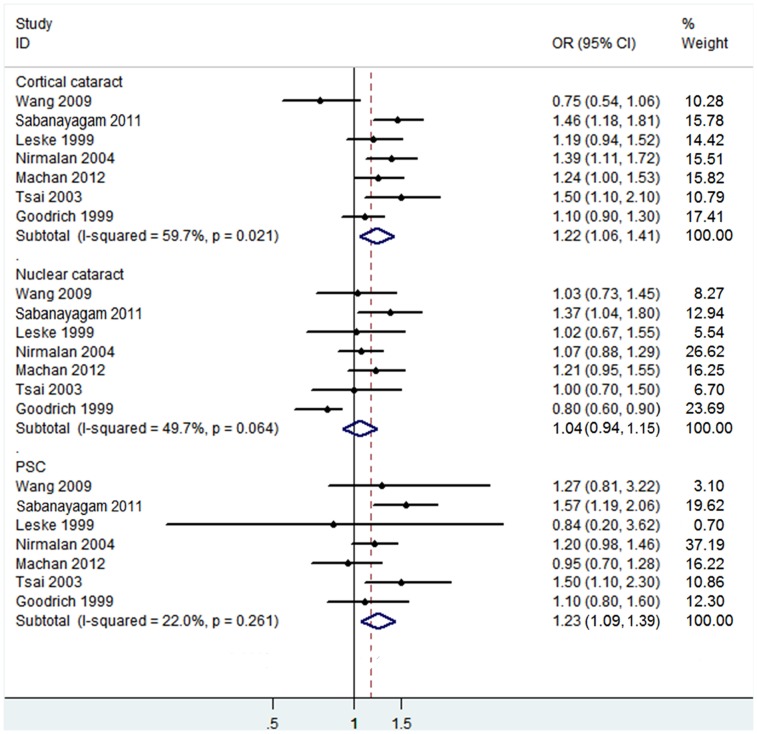
The association of hypertension with risk of cataract subtypes in case-control/cross-sectional studies.

Subgroup analysis was also conducted to investigate the effect of cataract subtypes on the relationship between hypertension and cataract risk (Figure 4). The pooled estimates indicated that hypertension significantly increased the risk of PSC (OR 1.23, 95% CI: 1.09–1.39; I^2^ = 22%, *P_ heterogeneity_* = 0.261) and the risk of cortical cataract (OR 1.22, 95% CI: 1.06–1.41; I^2^ = 59.7%, *P_ heterogeneity_* = 0.021), but didn’t increase the risk of nuclear cataract (OR 1.04, 95% CI: 0.94–1.15; I^2^ = 49.7%, *P_ heterogeneity_* = 0.064). No significant publication bias was found in those three subgroups (PSC: Begg’s *P* = 1.00, Egger’s *P* = 0.84; cortical cataract: Begg’s *P* = 1.00, Egger’s *P* = 0.62; nuclear cataract: Begg’s *P* = 0.76, Egger’s *P* = 0.65).

### Cohort Studies

9 studies from 8 articles were included in the meta-analysis of cohort studies (Table 2), of which 7 were included in the analysis of relationship between hypertension and any type of cataract risk; 6 were included in the subgroup analysis. All 9 studies were conducted among Caucasians. 7 studies were population-based, and 2 were hospital-based.

**Figure 5:**
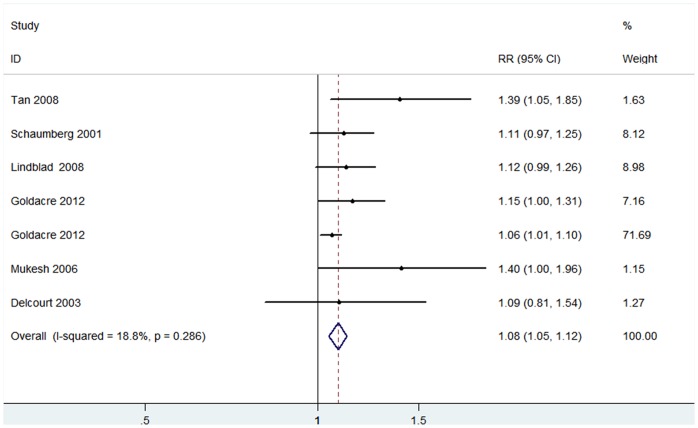
The association of hypertension with cataract risk in cohort studies.

The pooled RR indicated a significant increase of any type of cataract incidence in patients with hypertension in a fixed-effects model (RR 1.08, 95% CI: 1.05–1.12; I^2^ = 18.8%, *P_ heterogeneity_* = 0.286) (Figure 5). No publication bias was found among the 6 included studies (Begg’s P = 0.23, Egger’s P = 0.01).

**Figure 6:**
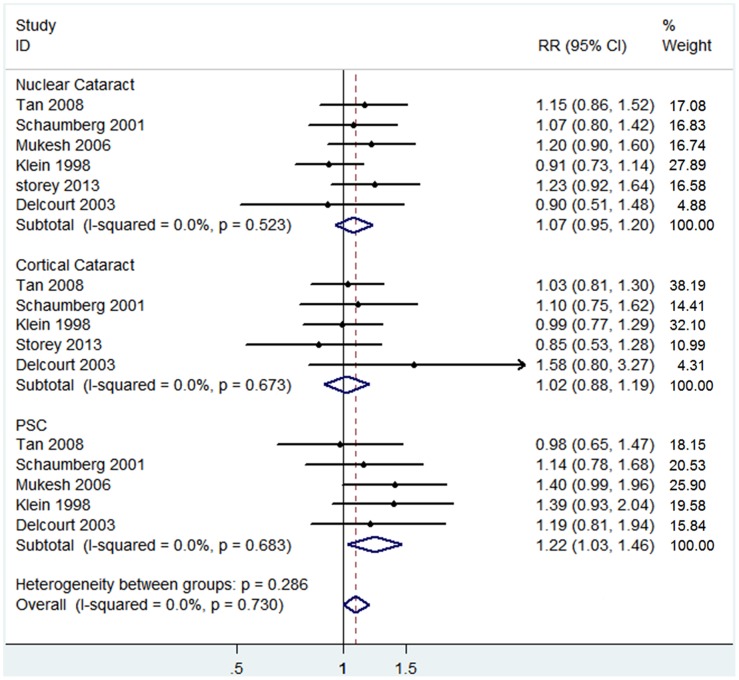
The association of hypertension with risk of cataract subtypes in cohort studies.

Figure 6 showed that 6 of 9 cohort studies afforded RR values with corresponding 95% CI for subgroup analysis. The outcome of subgroup analysis suggested that hypertension was significantly associated with PSC risk (RR 1.22, 95% CI: 1.03–1.46; I^2^ = 0%, *P _heterogeneity_* = 0.683). However, no evidence of a significant relationship between hypertension and cortical or nuclear cataract risk was found in the 6 included studies (cortical cataract: RR 1.02, 95% CI: 0.88–1.19; I^2^ = 0%, *P_ heterogeneity_* = 0.673; nuclear cataract: RR 1.07, 95% CI: 0.95–1.20; I^2^ = 0%, *P_ heterogeneity_* = 0.523) (Figure 6). No significant publication bias was detected in the three subgroups by means of Egger’s and Begg’s tests (PSC: Begg’s *P* = 0.46, Egger’s *P* = 0.31; cortical cataract: Begg’s *P* = 0.81, Egger’s *P* = 0.43; nuclear cataract: Begg’s *P* = 0.45, Egger’s *P* = 0.84).

## Discussion

The results of the present meta-analysis containing cohort and case-control or cross-sectional studies showed that hypertension was associated with an increased risk of cataract without regard for cataract types. It was true among both Mongolians and Caucasians. Besides, this association was demonstrated to be independent of the effect of pathoglycemia, obesity and dyslipidemia. An increased incidence of PSC related to hypertension was also revealed in both cohort and case-control or cross-sectional studies. There was no evidence of a significant relationship between hypertension and nuclear cataract. In terms of cortical cataract, the results from cohort studies conflicted with those from case-control and cross-sectional studies. But one must treat the pooled results in the subgroup analyses with caution due to the limited number of involved studies.

According to results reported by Sabanayagam et al. [28], people with severe hypertension have a higher risk of cataract than those with mild hypertension. Several studies indicated a linear positive correlation between blood pressure and cataract risk [45], [58], which is in accord with our results. Duration of hypertension is also an important factor, indicating a relationship between longer duration and increased cataract risk [11].

Many studies suggested that hypertension is linked to cataract development in part because of anti-hypertension medications. Cumming et al. [59] reported a significant association between cataract risk and potassium-sparing diuretics, which is biologically plausible, as this kind of anti-hypertension medications can disturb the electrolyte balance across the lens fiber membrane [60]. Several other studies indicated that exposure to beta-blockers can also promote cataract formation [46], [57]; which is supported by experimental studies demonstrating that the use of beta-blockers could elevate levels of intracellular cyclic adenosine monophosphate, thereby resulting in the modification of lens proteins [46], [61]–[62]. It is worth noting that protective roles against cataract development were also found. Results reported by Klein et al. [57], [63] showed that people taking thiazide diuretics or angiotensin-converting enzyme inhibitors (ACEI) have a decreased nuclear cataract risk. These protective effects may be neutralized by the pathogenic roles of hypertension, which is consistent with the results of our study that nuclear cataracts have no significant relationship to hypertension. It should be noted that the effects of anti-hypertension drugs and hypertension on cataracts might be conflated by each other.

Several pathophysiological pathways may be helpful to understand the mechanisms of cataract development promoted by hypertension. Bautista et al. [12], [13] have found that elevated plasma levels of IL-6 and TNF-α appeared in individuals with hypertension, which are closely related to intense systemic inflammation with enhanced levels of C-reactive protein and promote the development of cataract [14], [15]. Hypertension was reported to cause conformational changes in lens capsule [16], thereby interfering with the transportation of potassium ion in lens epithelial cells. And this pathological process finally leads to a disorder of lens short-circuit current that plays a protective role against cataract formation [64]–[65]. Results from Ornek et al. [66] showed that hypertensive patients would have a significantly higher level of nitrite in their cataractous lenses; the resulting nitric oxide plays an important role in the pathogenesis of human cataract. What’s more, Johnson et al. [67] reported a novel gene mutation related to both cataract and hypertension, which may be helpful in finding the potential fundamentals of genetics.

Heterogeneity was detected by means of Cochran’s Q statistic and I^2^ score among the studies included in this meta-analysis., which might be caused by different adjustments for confounders, the various ages of the study populations, different sample sizes, and various cataract criteria. We performed a meta-regression analysis to assess the effect of sample size, study design, study conducted race, publication year and cataract criteria on the heterogeneity, but none was identified as the main source of heterogeneity. The existence of heterogeneity indicates the need for unified methodologies in future studies.

Our meta-analysis has several strengths. Not only cross-sectional or case-control studies but also cohort studies were included in this analysis; the latter tends to be insusceptible to selection bias. Each study was adjusted for age, which is the most reliable independent risk factor for cataract [5]. Most studies included in our meta-analysis were based on the general population for more generalizable results. In addition, we performed a subgroup analysis to rule out the influences of pathoglycemia, obesity and dyslipidemia, which are thought to be the common risk factors for both hypertension and cataract [31], [33]_._

Potential limitations of our meta-analysis, which may affect the interpretation of results, should be mentioned. Firstly, the assessment of cataract and adjusted factors varied among the studies, contributing to an increase of heterogeneity. Secondly, the possibility of publication bias is of concern because studies without statistically significant results would not be published. Thirdly, only articles published in English-language journals were included, which might lead to language bias and the omission of inconclusive or negative studies in non-English articles. Fourthly, neither Egger’s linear regression test nor Begg’s rank correlation test played a perfect role in the present meta-analysis owing to an insufficient number of studies. Finally, the studies included in the subgroup analysis were too few to improve the accuracy of results.

In summary, our study showed that hypertension would increase cataract risk, and this association was independent of pathoglycemia, obesity and dyslipidemia. The results of subgroup analysis suggested a significant association between hypertension and PSC. These findings indicated that hypertension control would help to reduce cataract prevalence and related cataract surgery costs. To confirm these findings, further efforts should be made to make a better understanding of the potential biological mechanisms. Large-scale and long-term randomized controlled trials in various populations should be carried out in future studies to provide more powerful evidence.

## Supporting Information

Checklist S1 **PRISMA 2009 checklist in this meta-analysis.**
(DOC)

File S1 **The Newcastle-Ottawa Scale (NOS) for Assessing the Quality of Studies Included into Present Meta-Analyses.**
(DOC)
